# Short-term disturbance by a commercial two-dimensional seismic survey does not lead to long-term displacement of harbour porpoises

**DOI:** 10.1098/rspb.2013.2001

**Published:** 2013-11-22

**Authors:** Paul M. Thompson, Kate L. Brookes, Isla M. Graham, Tim R. Barton, Keith Needham, Gareth Bradbury, Nathan D. Merchant

**Affiliations:** 1 Lighthouse Field Station, Institute of Biological and Environmental Sciences, University of Aberdeen, Cromarty IV 11 8YL, UK; 2 Kongsberg Maritime Ltd, 11 The Briars, Waterberry Drive, Waterlooville, Hampshire PO7 7YH, UK; 3 WWT Consulting, Slimbridge, Gloucestershire GL2 7BT, UK; 4 Department of Physics, University of Bath, Bath, Somerset BA2 7AY, UK

**Keywords:** *Phocoena phocoena*, oil and gas exploration, acoustic disturbance, marine mammal conservation, marine spatial planning

## Abstract

Assessments of the impact of offshore energy developments are constrained because it is not known whether fine-scale behavioural responses to noise lead to broader-scale displacement of protected small cetaceans. We used passive acoustic monitoring and digital aerial surveys to study changes in the occurrence of harbour porpoises across a 2000 km^2^ study area during a commercial two-dimensional seismic survey in the North Sea. Acoustic and visual data provided evidence of group responses to airgun noise from the 470 cu inch array over ranges of 5–10 km, at received peak-to-peak sound pressure levels of 165–172 dB re 1 µPa and sound exposure levels (SELs) of 145–151 dB re 1 µPa^2^ s^−1^. However, animals were typically detected again at affected sites within a few hours, and the level of response declined through the 10 day survey. Overall, acoustic detections decreased significantly during the survey period in the impact area compared with a control area, but this effect was small in relation to natural variation. These results demonstrate that prolonged seismic survey noise did not lead to broader-scale displacement into suboptimal or higher-risk habitats, and suggest that impact assessments should focus on sublethal effects resulting from changes in foraging performance of animals within affected sites.

## 1. Introduction

Marine seismic surveys operate over extensive areas, producing some of the most intense man-made ocean noise [1,2]. Increasing awareness of the potential impacts of impulsive noise on marine mammals has led to the development of measures to minimize direct injuries in the near field, typically in the region of 500 m from seismic operations [3]. However, uncertainty over the extent to which protected species are displaced from favoured habitats remains a contentious issue for regulators of offshore energy developments [4].

Field studies of the impacts of seismic surveys on cetaceans have generally been limited to localized interactions with endangered baleen whale populations [5,6] or fine-scale responses of a few individuals to experimental or opportunistic exposure to airgun noise [7–9]. Attention has focused on impacts on baleen whale populations, because their low-frequency hearing suggests that they are most vulnerable to the effects of the low-frequency anthropogenic noise [10]. However, there is increasing concern over the extent to which expanding oil and gas exploration may affect other cetacean species in both temperate shelf seas [11] and arctic waters [12]. The only information available on behavioural responses of smaller cetaceans that have higher frequency hearing is based on observations from seismic survey vessels [13,14]. Although these observations include reports of aversive behaviour by small cetaceans [15], nothing is known about the spatial scales or longer-term consequences of these responses [1,16]. From a regulatory perspective, this is especially important, because potential impacts on protected species must increasingly be assessed in relation to longer-term population-level consequences [11]. Given increasing evidence of short-term responses to relatively low levels of noise [8,17], there are concerns that offshore energy developments could ensonify large areas, resulting in population impacts owing to displacement from preferred habitats [12].

Here, we investigated whether a commercial two-dimensional seismic airgun survey in the North Sea led to changes in the occurrence of harbour porpoises (*Phocoena phocoena*), a small cetacean that is widely distributed across northern shelf seas, and considered particularly sensitive to anthropogenic noise [2,18]. We used a broad-scale array of passive acoustic monitoring devices (C-PODs) and digital aerial surveys to detect changes in echolocation activity and porpoise sightings across a 2000 km^2^ area around the seismic survey. We aimed, first, to assess how changes in the occurrence of porpoises varied with distance from the seismic vessel and time since exposure. Second, we aimed to determine whether the seismic survey resulted in broader-scale displacement.

## 2. Material and methods

### (a) Seismic survey characteristics

**Figure 1.**
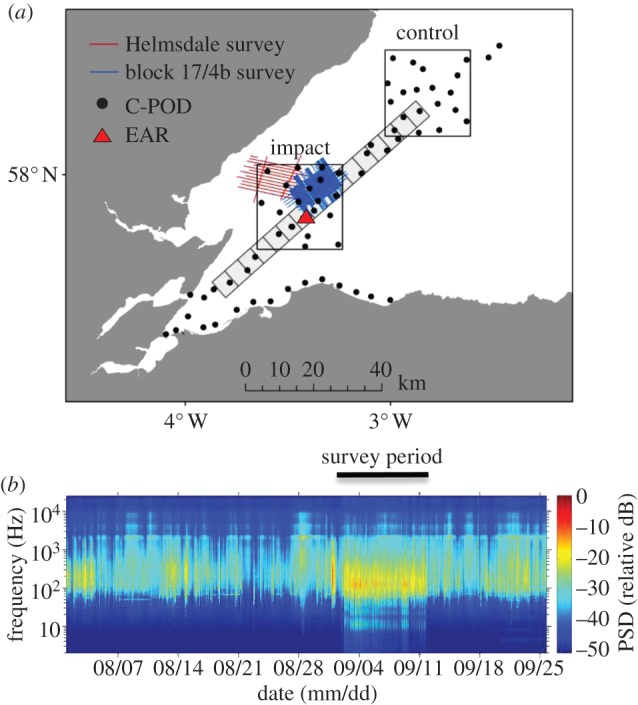
(*a*) Map of the study area showing the location of the 2011 seismic survey, C-POD sampling sites used in 2010 and 2011, the study's impact and control blocks, and the gradient of 5×5 km blocks used for the analysis of digital aerial survey data. (*b*) Spectrogram showing variation in received noise levels in the impact block recorded using the moored environmental acoustic recorder in August–October 2011. (Online version in colour.)

Seismic surveys were conducted over 10 days in two areas within the central Moray Firth, northeast Scotland (figure 1), using a 470 cu inch airgun array with a shot point interval of 5–6 s. Surveys were licensed by the Department of Energy and Climate Change (DECC), and followed the UK guidelines to reduce potential impacts on marine mammals [3]. See the electronic supplementary material, table S1 for details of the timing of seismic surveys.

Calibrated measurements of the airgun noise were made between 1 and 5 September 2011, at 15 sites between 1.6 and 61.8 km from the survey vessel (see electronic supplementary material, figure S1). Recordings were made from an 11.5 m workboat, using a RESON TC-4032 hydrophone, a RESON VP2000 conditioning amplifier and an NI USB-6251 16-bit analogue to digital convertor (National Instruments). The signal was sampled continuously at 500 000 samples per second and recorded onto a laptop computer. Water depths in the study area were typically less than 50 m, and recordings of received levels were measured at a depth of 10 m. Details of the equipment frequency response and calibration are provided in the electronic supplementary material.

An estimate of peak-to-peak sound pressure level (SPL) at 1 m from source was derived from far-field recordings made on 4 September as the seismic vessel passed within approximately 1660 m of the recording vessel; the closest distance at which recordings could be made without the system being overloaded. We considered the centre of the array as a point source 73 m behind the stern of the vessel, at a depth of 6 m, and estimated source levels by back calculating using a combination of parabolic (http://cmst.curtin.edu.au/products/actoolbox.cfm/) and ray-trace (http://oalib.hlsresearch.com/Rays/) models for low- and high-frequency components, respectively.

Safe thresholds for received SPLs are typically expressed on the decibel scale relative to a reference root mean square (r.m.s.) pressure of 1 µPa at 1 m [2], but this measure is highly dependent on the time window used for analysis when applied to pulsed noise sources such as seismic airguns [19]. We therefore followed suggested protocols for measuring pulsed sounds and present data using (i) peak-to-peak SPL in dB re 1 µPa and (ii) the SEL for single pulses in dB re 1 µPa^2^ s^−1^, using the region of the waveform that contained the central 90% of the pulse's energy [18,19]. For comparison with previous studies, we also present r.m.s. values for the region of the waveform that contained the central 90% of the pulse's energy.

Longer-term variation in relative noise levels at a site within the seismic survey area (57°53.7′ N 003°25.9′ W) was characterized by deploying a seabed mounted autonomous environmental acoustic recorder (EAR) [20] that recorded at 64 000 samples per second for 10 min in each hour between August and October 2011 (figure 1).

### (b) Passive acoustic monitoring

Harbour porpoises regularly echolocate [21], and we assume that variation in echolocation click detections provides an index of changes in the occurrence of harbour porpoises. Spatial and temporal variation in echolocation clicks was measured using v.0 and v.1 C-PODs (www.chelonia.co.uk), the digital successor of the T-POD that has been used extensively to study changes in the occurrence of harbour porpoises [22–24].

To assess how changes in porpoise occurrence varied with distance from the seismic vessel, we used a gradient design [25], with C-PODs deployed up to 70 km from the seismic vessel (figure 1). To determine whether there was a broad-scale impact over the whole survey period we also used a before-after-control-impact (B-A-C-I) design [26] with C-PODs deployed across 25 × 25 km impact and control blocks during August, September and October of 2010 and 2011 (figure 1).

In 2011, C-PODs were deployed at 70 sites in July, and devices with data were successfully recovered from 49 sites four months later. Baseline data were also collected in 2010, when C-PODs were deployed at 70 sites and 60 devices with data were recovered. Once recovered, data were downloaded and processed using v. 2.025 of the manufacturer's custom software to identify porpoise echolocation clicks with high, medium or low levels of confidence. Only click trains categorized with high or medium confidence were used in subsequent analyses [27].

Two metrics derived from these click train detections were used to compare spatial and temporal variation in the occurrence of porpoises. First, we determined the number of hours in each day that a porpoise click train was detected at each site; hereafter referred to as detection positive hours (DPHs) [28]. Second, sequences of click trains within each deployment were used to estimate the waiting time between a particular event and the next porpoise detection [22,29]. Waiting time was thus defined as *Δt*_p_: the time elapsed between *t*_p_ and *t*_detect_, where *t*_p_ was the time of the event and *t*_detect_ was the time of the first porpoise detection after *t*_p_. Previous visual and acoustic studies identified spatial variation in the density of porpoises across this study area in the absence of seismic activity [27,30]. We therefore used data from the week before the seismic survey to characterize baseline occurrence by producing a null distribution of waiting times between randomly selected observation times and the next porpoise detection for each of our C-POD sites. These could then be compared with waiting times following particular disturbance events (see below).

Echolocation detectors such as C-PODs can detect porpoises within a few hundred metres [31,32], but detection probability may vary either owing to slight differences in the sensitivity of individual devices or site-specific environmental conditions [29]. We minimized the influence of device variability by using the metrics DPH and waiting times, rather than finer scale measures such as the number of detection positive minutes per day or click trains per minute [27,28]. In addition, all analyses were based on relative changes within single C-POD deployments, using models that accounted for site-specific differences resulting either from differences in device sensitivity or underlying differences in the baseline occurrence of porpoises.

### (c) Aerial surveys

In 2011, digital aerial surveys were flown on 3 days before and 4 days during the seismic survey, using video techniques initially developed to survey seabirds [33]. Flights were made on days with suitable weather conditions (Beaufort sea state < 4, swell < 1.5 m, cloud base > 300 m), along a series of transects that provided a gradient of exposure to the airgun noise (see the electronic supplementary material, figure S2). Flight height and camera characteristics were standardized, so that the area within the video frame was known, allowing estimation of the relative density of porpoises. Data processing followed procedures established for birds [33], using trained analysts at Hi-Def Aerial Surveying Ltd (www.hidefsurveying.co.uk) to detect and georeference all objects from the video, and specialists at WWT Consulting Ltd to identify marine mammals and conduct standardized QA of all observations. Analyses were restricted to the 90% of small cetacean detections that were identified as either definite or probable harbour porpoises (see the electronic supplementary material, figure S3). Because aerial survey data collected during the seismic survey were pooled over 4 days, we estimated the relative density of porpoises in a series of 5 × 5 km blocks at increasing distance from the mean position of the vessel during these surveys (see figure 1 and the electronic supplementary material, figure S2).

In 2010, visual aerial surveys were made to provide an estimate of absolute density of porpoises within the study area using standardized line-transect sampling techniques [34]. We followed the established techniques from broader-scale porpoise surveys in the North Sea [35,36], using values of *g*(0) from the largest of these datasets [36] to calculate density from these data within program DISTANCE [34] (see the electronic supplementary material, figure S4 for further details).

### (d) Modelling short-term changes in porpoise occurrence

To assess the spatial scale of initial short-term responses to the airgun noise we calculated waiting times for each C-POD site from the first soft start at 15 : 15 GMT on 1 September. Distances to the seismic vessel were calculated from the vessel's GPS position at that time. Baseline occurrence at each site was characterized by randomly selecting 100 control points from the week prior to the seismic survey (23–30 August 2011), and calculating the waiting times from these points to the next porpoise detection. We then used generalized linear models to analyse the relationship between waiting times and distance, using a negative binomial error distribution to allow for overdispersion. For any given site, we would expect part of the waiting time (or all if distance had no effect) to be predicted from the baseline occurrence at that site, so models included the log-transformed median of the 100 randomly sampled waiting times for each site as an offset.

Within each 5 × 5 km block, the total area covered by digital aerial surveys made before and during seismic surveys was calculated from the length of survey line (based on the aircraft GPS trail) and camera strip width (based on flight height and camera specification). We then compared the density of porpoise sightings in each block in different periods.

### (e) Modelling changes in porpoise occurrence in relation to time since exposure

The extent of any displacement following exposure was investigated by estimating waiting times following the point of closest approach during those occasions when the seismic vessel passed within 5 km of a C-POD site while firing airguns. We excluded those occasions when the vessel returned to the site within an hour (based on average baseline waiting times at these sites). Each observed waiting time was then paired with a random waiting time from the same site in the week prior to the seismic survey, and a paired Wilcoxon test was used to compare distributions. We then used a mixed modelling approach to explore whether minimum distance of approach, time since the start of the seismic survey or number of consecutive approaches influenced waiting times. The model was built using the gamm function in the mgcv library [37] using linear predictors and a negative binomial error structure. The median value of the 100 random waiting times for each site was used as an offset variable.

### (f) Modelling broad-scale displacement

Broad-scale variation in porpoise occurrence was explored using data from a subset of sites in the impact (*n* = 12) and control (*n* = 6) blocks where data were available from 1 August to 23 October in both 2010 (no seismic survey) and 2011 (seismic survey). To avoid confounding effects of variation in device sensitivity (see above), our formal B-A-C-I analysis was restricted to single deployments in 2011, using data from August as our before time period and data from 2 to 11 September as the during time period. In 2011, data from 13 sites in the impact block and seven sites in the control block were available to use in a generalized linear mixed (GLM) model with a Poisson family error structure to account for non-negative integer values. C-POD site was included as a random intercept, which removed patterns in the residuals and improved the fit of the model. The fixed effects of the model were block and period and, crucially, an interaction term between these effects, the significance of which was used to detect whether or not there was an impact of seismic survey. Analyses were carried out in R v. 2.15.

## 3. Results

The seismic surveys were conducted between 1 and 11 September 2011, and produced peak-to-peak source levels that were estimated to be 242–253 dB re 1 µPa at 1 m. Individual survey lines of 7–15 km took 75–150 min to complete, resulting in regular noise exposure over a 200 km^2^ area throughout the 10 day survey period (see figure 1 and the supplementary electronic material, table S1).

**Figure 2.**
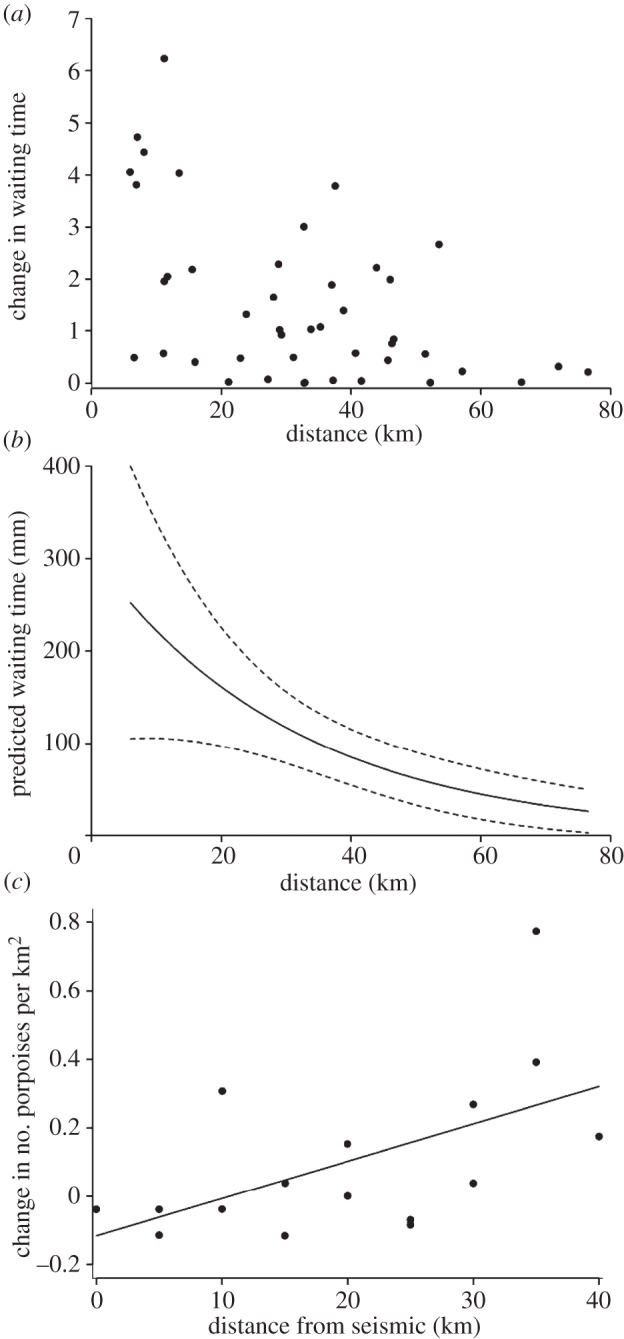
Changes in the occurrence of harbour porpoises in relation to distance from the seismic vessel. (*a*) Ratio of observed to baseline waiting times after the first airgun activity. (*b*) Predicted waiting times after initial exposure (solid line) and 95% confidence intervals (dashed lines) from a GLM, standardized for the median baseline waiting time of 84 min. (*c*) Observed changes in the relative density of porpoises from digital aerial surveys carried out before and during seismic surveys. Points are original data for each 5×5 km survey block (see the electronic supplementary material figure S1), the solid line is the linear model fit (*y* = 0.011*x* − 0.115).

**Figure 3.**
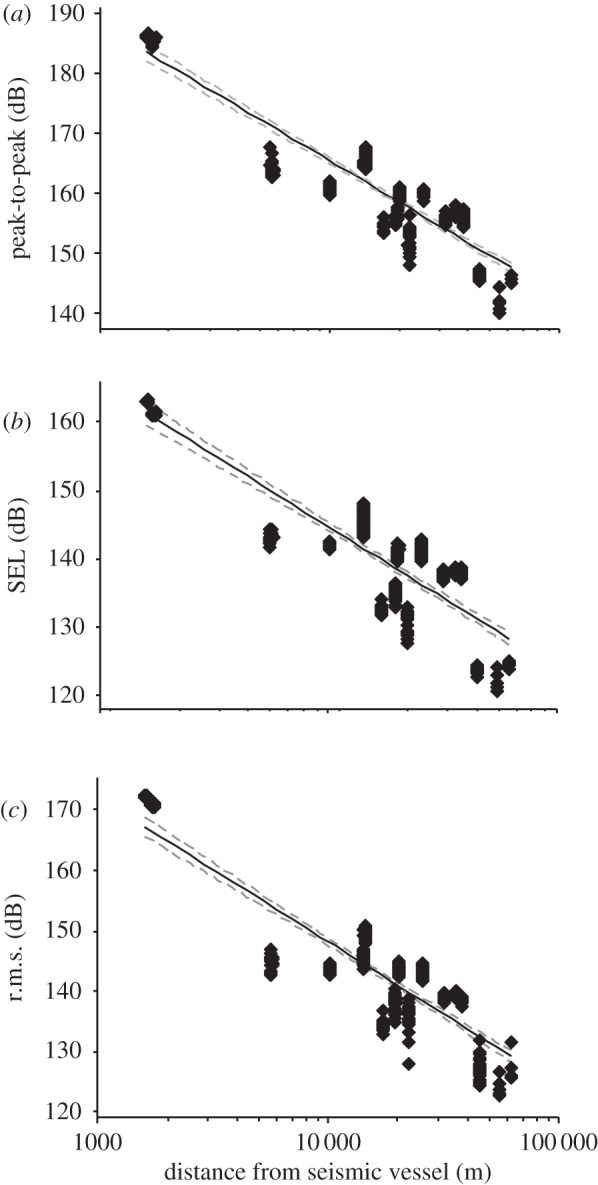
Variation in received noise levels at different distances from the seismic airgun array, expressed (*a*) as peak-to-peak SPL (dB re 1 µPa); (*b*) as SEL (dB re 1 µPa^2^ s^−1^) integrated over the central 90% of each pulse and (*c*) as r.m.s. (dB re 1 µPa) integrated over the central 90% of each pulse. Equations for the fitted logarithmic spreading loss are SPL = 255.77–22.6 log (range) (*F*_1,366_ = 1245.5, *p* < 0.001, *r*^2^ = 0.77); SEL = 227.95–20.8 log (range) (*F*_1,366_ = 716.55, *p* < 0.001, *r*^2^ = 0.66); r.m.s. = 244–23.97 log (range) (*F*_1,366_ = 1003.0, *p* < 0.001, *r*^2^ = 0.73).

Following the start of seismic surveys on 1 September, observed waiting times increased relative to baseline (figure 2*a*), indicating that there was an initial response to the noise, but that this effect diminished with distance from source (negative binomial GLM: *χ*^2^ = 10.2, d.f. = 1, *p* = 0.001; figure 2*b*). Using passive acoustic methods alone, such changes could reflect either individual movement or a change in vocalization rate [38]. However, comparison of detection rates of porpoises from digital aerial surveys made before and during the seismic survey showed that the relative density of porpoises decreased within 10 km of the survey vessel and increased at greater distances (GLM: *F*_1,14_ = 6.28, *p* < 0.05; figure 2*c*), confirming that seismic operations resulted in short-term avoidance movements. Calibrated noise measurements made along this same impact gradient indicated that received peak-to-peak SPLs in the region 5–10 km from source varied from 165 to 172 dB re 1 µPa, whereas SELs for a single pulse were 145–151 dB re 1 µPa^2^ s^−1^, and r.m.s. levels were 148–155 dB re 1 µPa (figure 3).

**Figure 4.**
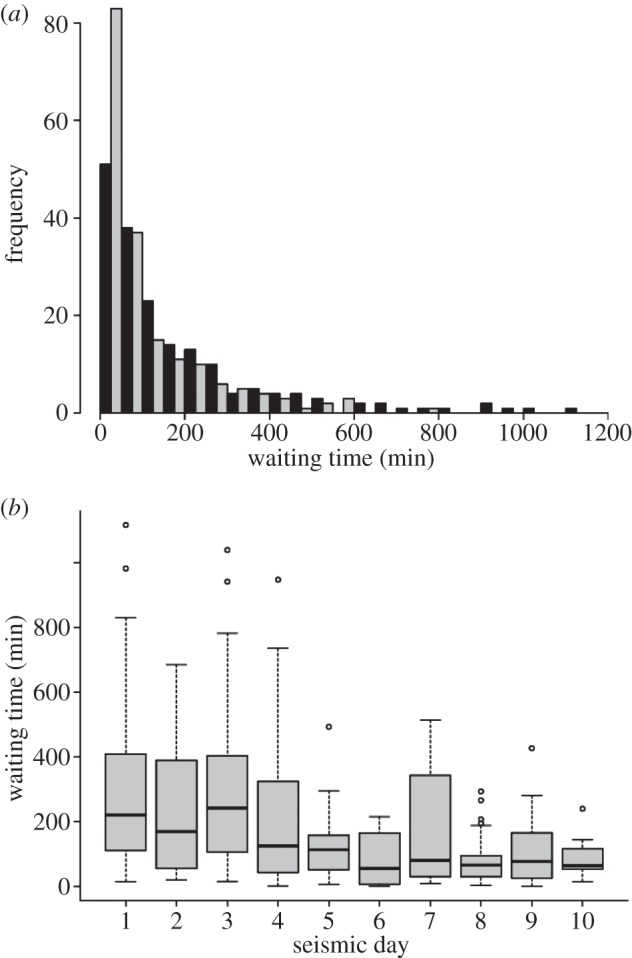
(*a*) Distribution of waiting times until the next porpoise detection following a close approach of the seismic vessel (black bars) in comparison to random points in the week before the survey (grey bars). (*b*) Shows the modelled reduction in these waiting times through the seismic survey campaign, with day 1 representing 2 September 2011.

The seismic vessel was firing airguns as it passed within 5 km of a C-POD site on 181 occasions. The frequency distribution of waiting times following these occasions show that porpoises were detected again at all sites within 19 h (median = 183 min), but that this was significantly longer than matched random waiting times (median = 57 min) from the week before the seismic survey (figure 4*a*; Wilcoxon test, *V* = 10907.5, *p* < 0.001). A decrease in waiting times through the 10 day seismic survey suggested that responses to this disturbance declined with increased exposure (figure 4*b* and table 1).

**Table 1.** Results of the generalized linear mixed model of waiting times to next acoustic detection of porpoises following a close approach by the seismic vessel. A negative binomial distribution was used and the random effect was site.

	estimate	s.e.	*p*-value
intercept	0.6452	0.2471	0.010
days since start of seismic survey	–0.0675	0.0283	0.018
number of consecutive approaches	0.3933	0.0751	<0.001

**Figure 5.**
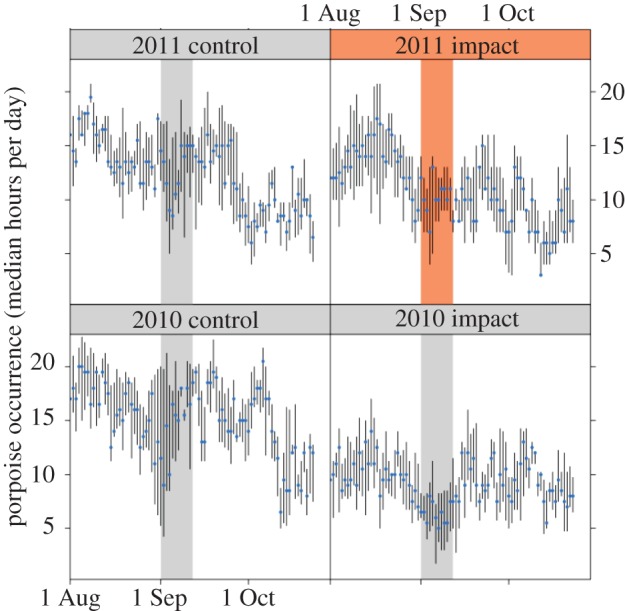
Variation in the median number of hours (with interquartile ranges) that porpoises were detected on C-PODs in the impact and control blocks in the summers of 2011, when the seismic survey was carried out, and in the previous baseline year. The timing of the seismic survey is depicted by dark shading in the panel for the impact block in 2011 and equivalent periods are lightly shaded in the other panels. (Online version in colour.)

Analysis of porpoise detections through the three-month period that centred on the seismic survey demonstrated consistently high levels of porpoise occurrence in impact and control areas in both 2011 and 2010, with evidence of seasonal and interannual variability (figure 5). Our assumption that variations in acoustic detections provide an index of underlying changes in density in these areas was supported by data collected in 2010, when different rates of acoustic detections in the control and impact area reflected absolute estimates of density obtained from visual aerial surveys (table 2). In 2011, observed seasonal declines in occurrence resulted in reductions in acoustic detections in both impact and control areas during the seismic survey, as shown by the significant effect of period in table 3. There was also a significant difference between blocks (table 3), with higher detections in the control block (figure 5). In addition, our B-A-C-I analysis using 2011 data identified a significant impact of the seismic survey, as shown by the interaction term in table 3. However, the effect size was only small, with a reduction in porpoise detections of 16.7% (to a median of 10 h per day) in the impact block compared with a reduction of 12.5% (to a median of 14 h per day) in the control block (figure 5).

**Table 2.** Comparison of acoustic detections (from C-PODs) and line-transect estimates of absolute density of porpoises (from visual aerial surveys) in the impact and control areas in August and September of 2010, the year before the seismic survey. Density estimates are presented as the number of individual porpoises per km^2^.

area	acoustic estimates				direct estimates	
	detection +ve hours per day		waiting times (min)			
	median	IQ range	median	IQ range	density	95% CI
impact	9	6–12	65	28–152	0.50	0.36–0.68
control	14	10–18	42	21–88	0.75	0.38–1.48

**Table 3.** The results of a Poisson generalized linear mixed model used to investigate the effect of a seismic survey on acoustic detection of porpoises, before (1–31 August 2011) and during (2–11 September 2011) the survey in the control and impact block.

	estimate	s.e.	*p*-value
intercept	2.721	0.090	<0.001
block	−0.224	0.112	0.044
period	−0.143	0.037	<0.001
block: period interaction	−0.102	0.048	0.035

## 4. Discussion

Fine-scale tracking of a few individual large cetaceans has previously detected behavioural responses at noise levels below thresholds used in the US to identify potential harassment to cetaceans [8,17], and studies of baleen whales on localized foraging grounds [5] and migration routes [39] also detected fine-scale behavioural responses to seismic vessel noise. Captive porpoises exposed to airgun noise exhibited aversive behavioural reactions at peak-to-peak SPL above 174 dB re 1 µPa, and an SEL of 145 dB re 1 µPa^2^ s^−1^ [18]. Our data indicate that animals were exposed to similar levels of received noise within 5–10 km of the seismic vessel, resulting in avoidance movements. Similar results have been reported from studies of harbour porpoise responses to other impulsive noise sources such as pile-driving around offshore wind farms [24,40,41]. These passive acoustic techniques are unable to detect individual movements, so we were unable to confirm whether or not the same animals returned to impacted sites. Nevertheless, our data on these group responses show that either these or other individuals returned to impacted areas within a day (figure 4). Furthermore, while a significant decrease in occurrence was detected over the entire seismic survey period (table 3), this effect was small in relation to natural variation, and porpoises continued to occur at sites within the impact study block for around 10 h per day even during the seismic survey (figure 5).

Responses to anthropogenic noise are expected to vary in relation to both the species of marine mammal [2] and context [42], and additional work is now required to assess the generality of our findings. Nevertheless, our focus on harbour porpoises makes these results relevant to the management of Northern Hemisphere shelf seas, as this is the most common cetacean in many areas currently or potentially exposed to offshore energy developments [11]. On the one hand, this species’ relatively high sensitivity to anthropogenic noise may provide a conservative indication of the level of response by other small cetaceans using these areas [2,18]. However, like many other parts of the North Sea, our study area has a long history of exposure to impulsive noise and other anthropogenic activity [11,43]. In combination with our evidence for a decrease in response levels over the 10 day seismic survey (figure 4*c*), it seems likely that stronger responses may be expected in populations that have previously had little exposure to anthropogenic noise [12]. Similarly, source levels from this two-dimensional shallow hazard survey were of lower magnitude than some large-scale seismic surveys. For example, deep penetration three-dimensional surveys may use airgun arrays of several thousand cubic inches, with source levels of up to 265 dB re 1 µPa [44], potentially eliciting stronger responses in the near field.

Among baleen whales, modification of song characteristics in the presence of seismic survey noise [10] suggests that displacement from ensonified areas might be a direct response by animals to reduce masking of communication calls. This is unlikely to be a factor affecting observed responses in harbour porpoises and other small cetaceans, because most of the energy from seismic airguns is well below the frequencies used by these species to communicate [45,46]. We cannot rule out the possibility that the observed responses by harbour porpoises were an indirect response to the noise, mediated through changes in prey behaviour [47]. It is also possible that animals perceived the noise as annoying, which could lead to displacement [48]. Alternatively, aversive responses to anthropogenic noise in small cetaceans may reflect an anti-predator response [49], with the level of response resulting from a trade-off between fear and the costs of moving to different habitats [50]. Harbour porpoises have high energy demands compared with other small cetaceans [51] and, like small passerine birds, may therefore be constrained to return rapidly to high-quality feeding patches under even relatively high predation risk [52]. This highlights the possibility that the extent to which harbour porpoises may be displaced by long periods of impulsive noise could vary in relation to habitat quality. Density estimates in our study area (table 2) were comparable with those recorded in high density areas within the North Sea [35], suggesting that our study area represented relatively high-quality porpoise habitat. Longer-term displacement may therefore be more likely following industrial activity in marginal habitats [29].

Mitigation of the potential impacts of anthropogenic noise on cetaceans focuses on reducing near-field injuries [3], and risk assessments are based on the assumption that animals flee from loud noise sources. To a certain extent, our results support this assumption, but we also observed declines in the response to airgun noise during the survey period. This decline in response could have resulted either from habituation or tolerance to airgun noise, meaning that one cannot assume that the outcome of the disturbance is neutral [53]. In some development areas, there are concerns that animals could be exposed to an increased risk of mortality should they be displaced from high-quality habitats [12], or into areas where there was a higher risk of by-catch [54] or interspecific competition [55]. Our evidence of continued use of areas impacted by noise from a seismic survey provides a clearer focus for the assessments of population consequences of acoustic disturbance that are increasingly required to support development proposals [11]. These findings suggest that broader-scale exclusion from preferred habitats is unlikely. Instead, individual fitness and demographic consequences are likely to be more subtle and indirect, highlighting the need to develop frameworks to assess the population consequences of sublethal changes in foraging energetics of animals occurring within affected sites [1,56].

## Data accessibility

Data available from the dryad repository: http://doi.org/10.5061/dryad.hv4db.
